# Genome Sequence of Genotype 1A Hepatovirus A Isolated from Plasma from a Haitian Child

**DOI:** 10.1128/mra.00449-22

**Published:** 2022-08-11

**Authors:** M. Mahbubul Alam, Maha A. Elbadry, Julia C. Loeb, Caroline J. Stephenson, Rigan Louis, Carla Mavian, Remi Charrel, Sabita Rezwana Rahman, J. Glenn Morris, John A. Lednicky

**Affiliations:** a Emerging Pathogens Institute, University of Florida, Gainesville, Florida, USA; b Department of Environmental and Global Health, College of Public Health and Health Professions, University of Florida, Gainesville, Florida, USA; c State University of Haiti Faculty of Medicine and Pharmacy, Port-au-Prince, Haiti; d Department of Pathology, Immunology, and Laboratory Medicine, College of Medicine, University of Florida, Gainesville, Florida, USA; e Unite des Virus Emergents, Aix Marseille Université, IRD 190, INSERM U1207, Marseille, France; f University of Dhaka, Dhaka, Bangladesh; g Department of Medicine, College of Medicine, University of Florida, Gainesville, Florida, USA; Portland State University

## Abstract

Genotype 1A hepatovirus A was identified by quantitative reverse transcription-PCR and isolated from plasma from a Haitian child with acute undifferentiated febrile illness and malaise. The strain was most closely related to Brazilian strains, consistent with recognized patterns of virus movement in the Caribbean region.

## ANNOUNCEMENT

Hepatovirus A (HAV) is a single-stranded nonenveloped RNA virus belonging to the genus *Hepatovirus* of the family *Picornaviridae*. In low- and middle-income countries, it has been estimated that 90% of children are infected with the virus before the age of 10 years (1). As reported previously, our group monitored the occurrence of acute undifferentiated febrile illness in a cohort of children in Gressier, Haiti, from 2014 to 2019 (2–4). Studies were approved by the University of Florida and Haitian national institutional review boards, and signed informed consent forms from parents and assent from students were obtained before enrollment in the study.

HAV genomic RNA was detected by quantitative reverse transcription-PCR (qRT-PCR) in 2 (0.3%) of 677 plasma samples collected from febrile children as part of this study, by using the primer system described by Jothikumar et al. (5) and a modified HAV probe, namely, 5′-6-carboxyfluorescein (FAM)-CTTARGCTARTACTTCTATGAAGAGATGC-black hole quencher 1 (BHQ1)-3′, in which two R degeneracies (underlined) were inserted to replace a G at position 417 and an A at position 422. Primers and probe were combined and freeze-dried in a single glass vial (6). The quantification cycle (*C_q_*) values for sample 15-1-1251, which was collected in January 2015, and sample 18-1-2097, which was collected in November 2016, were 33.37 and 40.27, respectively. Attempts were made to isolate the virus in MRC-5 cells (7) and were successful only for sample 18-1-2097. Sample 18-1-2097 was from a 6-year-old child who presented with a temperature of 38.1°C and complaints of malaise and mouth sores; no jaundice was noted.

The genomic sequence of sample 18-1-2097 was obtained from virus RNA that had been extracted from plasma (8) and Sanger sequenced with a gene-walking approach (9, 10) using nonoverlapping primers. Briefly, viral RNA was extracted from 140 μl of plasma using a QIAamp viral RNA extraction kit (Qiagen, Valencia, CA), RT was performed using the AccuScript high fidelity first strand cDNA kit (Agilent Technologies, Santa Clara, CA) in the presence of SUPERase-In RNase inhibitor (Ambion, Austin, TX), and PCR was sequentially performed using Q5 high fidelity DNA polymerase (New England Biolabs, Inc., Ipswich, MA) and the primers identified in Table 1. To determine the sequence of the 5′ end of the virus genome, 20 μL of purified RNA was treated with DNase- and RNase-free proteinase K (New England Biolabs) to remove the 5′ VPg (11–13), followed by 5′ rapid amplification of cDNA ends (RACE) using a FirstChoice RLM-RACE kit (Thermo Fischer Scientific) following the manufacturer’s instructions. Quality scores for the Sanger sequences ranged from 52 to 67 (11–13). Excluding the poly(A) tail, the virus genome length is 7,477 ribonucleotides (rnt) (A, 2,181 rnt; U, 2,449 rnt; G, 1,638 rnt; C, 1,209 rnt), with a G+C content of 38.1%.

**TABLE 1:** Primers for sequencing HAV

Primer	Sequence (5′ to 3′)	Nucleotide positions in GenBank accession no. OK625565.1	Reference	Nucleotide positions in GenBank accession no. MG049743.1	Amplicon size (bp)
5′-RACE-R	AACAACTCACCAATATCCGC	480–461	2		
HAV for	GGTAGGCTACGGGTGAAAC	392–410	2		88
HAV rev	AACAACTCACCAATATCCGC	480–461	2		
HAV For4	TACCTCACCGCCGTTTGCCTAGGC	64–87	1		417
HAV rev	AACAACTCACCAATATCCGC	480–461	2		
HepA for1	CTTAAGCTATTACTTCTATGAAGAGATGC	413–441	This work		648
HepA rev1	CAGCTTCACCACATCCAATTTTGCAACTTC	1060–1031	This work	1030–1001	
HepA for 2	GGCTCACTACACATGCTCTCTTTCATG	1005–1031	This work		853
HepA rev 2	CTACCTGAATGATATTTGGTTGGAAAAACC	1857–1828	This work	1827–1798	
HepA for 3	GGCTTCTATCTGTCAAATGTTTTG	1771–1794	This work		810
HepA rev 3	GATGGTAAACCATGCGGAGGATTTGAAG	2580–2553	This work	2550–2523	
HepA for 4	GGGAAGGTCTCACTTTTTGTG	2473–2493	This work		847
HepA rev 4	CCTAGTATCAGCAGTTACTCCTCTCC	3319–3294	This work	3289–3264	
HepA for 5	GTGCTTCCACCTCCTAGGAAAATGAAG	3200–3226	This work		952
HepA rev 5	GCTGGTTATCTTTAAGAATGTTAAG	4151–4127	This work	4121–4097	
HepA for 6	GGTTATATACCAAATTGAAGGAT	4068–4090	This work		816
HepA rev 6	CACAAGACATGTCCTTGATTGCATC	4883–4859	This work	4853–4829	
HepA for 7	GATCGTAGACTTCATTTTAAGGTTGAAG	4763–4790	This work		843
HepA rev 7	CTTGATAAAATGTTGAGTAATATCTCT	5605–5579	This work	5575–5549	
HepA for 8	GGGATTTCAAGATGTTGTTCTAATG	5530–5554	This work		844
HepA rev 8	GGCCAGTCGTGGATGAACTCCTAACAG	6373–6347	This work	6343–6317	
HepA for 9	GGCTCCAGGCATTGATGCTATTAATA	6241–6266	This work		773
HepA rev 9	GAACATCTCTGGAAAAGACTAT	7013–6992	This work	6983–6962	
HepA for 10	GAGGATTCTTTGTTACGGAGATG	6961–6983	This work		542
HepA rev 10	TTTTTTTTTTTTTTTTTTTTTTTTTA	7502–7477	This work		

**FIG 1:**
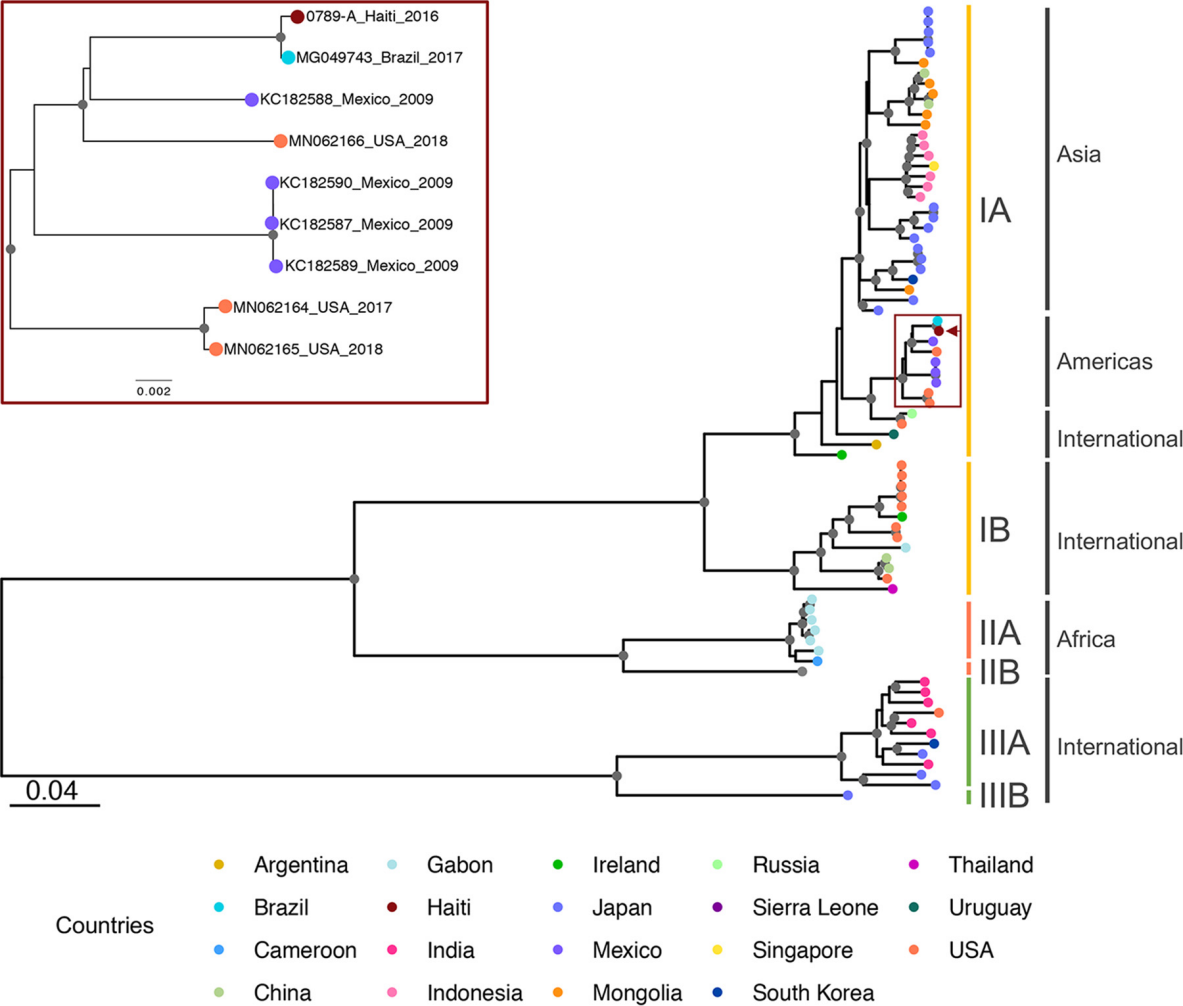
Phylogenetic inference of the HAV strains from a human source. The maximum likelihood phylogenetic tree of HAV strains from humans is based on the complete genome and was inferred using IQ-TREE. Gray circles at internal nodes represent >90% bootstrap support. Colored circles at the tips show the collection locations based on the legend at the bottom. The red rectangle shows the magnification of the subtree based on the American subclade containing the isolates from Haiti, Brazil, Mexico, and the United States with GenBank accession numbers (rectangle in the full phylogenetic tree). The Haitian isolate is indicated with a red arrow. Genotype classification is shown to the right of the tree.

The maximum likelihood phylogeny was constructed by using IQ-TREE (14–19) with all available complete genomes for HAV strains from humans available in GenBank; genotyping followed the methods described by Ramachandran et al. (20). The phylogenetic analysis shows that the genome sequence of the Haitian HAV isolate belongs to a well-supported monophyletic clade in genotype 1A that includes HAV genomes from the Americas, including Brazil, Mexico, and the United States, between 2009 and 2018 (Fig. 1). In particular, the genome sequence of the Haitian isolate clusters near a Brazilian HAV genome from 2017; the phylogenetic proximity and the short branches separating the Haitian genome sequence from the Brazilian sequence, which was from an HAV case that occurred 1 year after the collection of the virus in Haiti, are consistent with a recent common source or exchange of viruses between these two countries.

### Data availability.

The virus was designated hepatovirus A/0789/Haiti/2016, and its sequence was deposited in GenBank under accession number OK625565.1.
